# Editorial Comment from Dr Mori to Association between antibiotic use and subsequent risk of prostate cancer: A retrospective cohort study in South Korea

**DOI:** 10.1111/iju.15402

**Published:** 2024-01-24

**Authors:** Keiichiro Mori

**Affiliations:** ^1^ Department of Urology The Jikei University School of Medicine Tokyo Japan; ^2^ Department of Urology Medical University of Vienna Vienna Austria

Prostate cancer is recognized as not only the most common of all solid cancers but also the second most common cause of cancer‐related death in men.^1^ Several studies suggest that antibiotic use may affect overall cancer incidences, but the association between antibiotics and prostate cancer remains still unclear.^2^, ^3^

In this issue of the *International Journal of Urology*, Park and Hong et al. assessed the association between the use of antibiotics and their associated risk of prostate cancer by drawing on the Korean National Health Insurance Service (NHIS) database, which accounted for over 1 000 000 patients and concluded that long‐term use of antibiotics may affect the incidence of prostate cancer.^4^ This is the first retrospective study evaluating the association between antibiotic use and prostate cancer in Asia and sheds new light on the long‐term use of antibiotics and associated risk of developing prostate cancer in Asian populations.

However, this study has several major limitations that are worth noting. First, the antibiotics evaluated vary considerably in their effects. Of note here is the report of Charalabopoulos et al., which suggested that different types of antibiotics have different levels of penetration into the prostate.^5^ In this study, antibiotic agents including macrolides, penicillins, cephalosporins, fluoroquinolones, and tetracyclines increased the risk of prostate cancer with statistical significance under the *p*‐value <0.05. Given little or no evidence of a direct relationship shown between their penetration levels and associated risk of prostate cancer, however, further studies are needed to investigate this presumed relationship, as well as the accurate, in‐depth mechanisms involved, which likely account for the differences in their association with the risk of prostate cancer. Second, they were unable to include for analysis of the PSA level in the participants, despite being one of the most promising indicators for detecting prostate cancer, due to the limitations of the database drawn on. Thus, while this paper provides valuable information, the results reported should be interpreted with great caution.

## CONFLICT OF INTEREST STATEMENT

The author has nothing to declare.
